# A sea-level plateau preceding the Marine Isotope Stage 2 minima revealed by Australian sediments

**DOI:** 10.1038/s41598-019-42573-4

**Published:** 2019-05-10

**Authors:** Takeshige Ishiwa, Yusuke Yokoyama, Jun’ichi Okuno, Stephen Obrochta, Katsuto Uehara, Minoru Ikehara, Yosuke Miyairi

**Affiliations:** 1 Atmosphere and Ocean Research Institute, The University of Tokyo, 5-1-5 Kashiwanoha, Kashiwa, Chiba, 277-8564 Japan; 2 Department of Earth and Planetary Science, Graduate School of Science, The University of Tokyo, 7-3-1 Hongo, Bunkyo-ku, Tokyo, 113-0033 Japan; 3 National Institute of Polar Research, 10-3 Midoricho, Tachikawa, Tokyo, 190-8518 Japan; 4 Graduate School of International Resource Science, Akita University, 1-1 Tegata-Gakuenmachi, Akita, Akita, 010-8502 Japan; 5 Research Institute for Applied Mechanics, Kyushu University, 6-1 Kasugakoen, Kasuga, Fukuoka, 816-8580 Japan; 6 Center for Advanced Marine Core Research, Kochi University, B200 Monobe, Nankoku, Kochi, 783-8502 Japan; 7 Present Address: National Institute of Polar Research, 10-3 Midoricho, Tachikawa, Tokyo, 190-8518 Japan

**Keywords:** Palaeoceanography, Palaeoclimate

## Abstract

Further understanding of past climate requires a robust estimate of global ice volume fluctuations that in turn rely on accurate global sea-level reconstructions. An advantage of Marine Isotope Stage 2 (MIS 2) is the availability of suitable material for radiocarbon dating to allow comparison of sea-level data with other paleoclimatic proxies. However, the number and accuracy of sea-level records during MIS 2 is currently lacking. Here we present the history of MIS 2 eustatic sea-level change as recorded in the Bonaparte Gulf, northwestern Australia by reconstructing relative sea level and then modeling glacial isostatic adjustment. The isostatically-corrected global sea-level history indicates that sea-level plateaued from 25.9 to 20.4 cal kyr BP (modeled median probability) prior reaching its minimum (19.7 to 19.1 cal kyr BP). Following the plateau, we detect a 10-m global sea-level fall over ~1,000 years and a short duration of the Last Glacial Maximum (global sea-level minimum; 19.7 to 19.1 cal kyr BP). These large changes in ice volume over such a short time indicates that the continental ice sheets never reached their isostatic equilibrium during the Last Glacial Maximum.

## Introduction

Global sea-level change during the Pleistocene glacial-interglacial cycles reflects fluctuating continental ice volume that is primarily related to the variable planetary distribution of incoming solar radiation caused by changes in the Earth’s orbit^1,2^. Marine Isotope Stage 2 (MIS 2; 29,000–14,000 years ago^3^), the latter portion of the most recent glaciation, includes the Last Glacial Maximum (LGM), during which ice sheets reached their largest volume^4^. A number of paleoclimate records span MIS 2, facilitating comparison of climate proxy data to sea-level reconstructions^5^. However, reconstructions from far-field sites, locations distal from past ice sheets such as Barbados^6,7^, the Sunda Shelf^8,9^, the Huon Peninsula^10–12^ and the Bonaparte Gulf^13–15^, provide conflicting information with respect to the amplitude and timing of the LGM. Subaerial exposure during ice-sheet expansion and sea-level regression followed by sea-level rise complicates pre-LGM sea-level reconstruction, contributing to this discrepancy.

**Figure 1:**
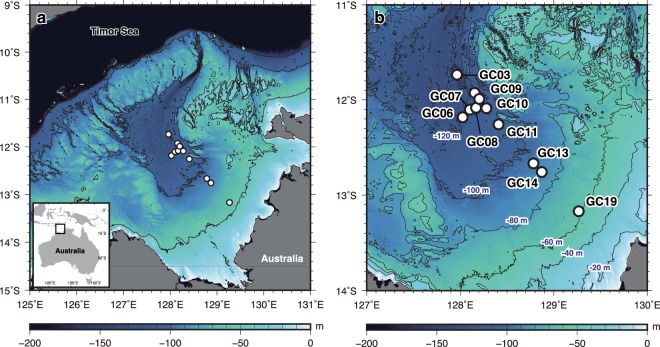
KH11-1 core locations and the topography of the Bonaparte Gulf. (**a**) Map of the Bonaparte Gulf. Contour interval is 50 m. White circles are coring sites occupied during R/V Hakuho-Maru cruise KH11-1. (**b**) Enlarged portion of panel a. Contour interval is 20 m. White circles are the core locations. The location of core RS176-GC5^13,14^, which records the LGM sea-level lowstands as the brackish/estuarine environment, is the same as KH11-1-GC06. Bathymetric data is from Geoscience Australia^51^. Figures were drawn using The Generic Mapping Tools^52^.

The Bonaparte Gulf (Fig. 1), northwestern Australia, is a broad and shallow continental shelf with a centered depression surrounded by carbonate platforms and terraces. These platforms and terraces are presently submerged but were exposed when sea level fell 70 m below present levels. During this time, connection to the Timor Sea was preserved through the presence of incised valleys^16,17^. The Bonaparte Gulf is a far-field site and one of the few sites with high-resolution relative sea-level (RSL) records spanning MIS 2^13–15^. The onset of the LGM, as recorded in the Bonaparte Gulf, occurred at ca. 21 cal kyr BP (calendar kiloyears ago before AD 1950), with RSL reaching −125 ± 4 m and terminating at 19 cal kyr BP with a rapid sea-level rise, the so-called “19 ka event”^13–15^. High-resolution micropaleontological analyses indicate that the appearance of brackish/estuarine conditions is constrained to within ±2 m^13,14^. However, further work from a transect of marine core sediments is needed to detect the timing of changes in shallow and marginal marine facies, which are also important sea-level indicators^18,19^.

A large tidal range with an amplitude of close to 6 m, comparable to the sea-level equivalent ice volume of the modern Greenland Ice Sheet, is presently observed in the near-shore region of the Bonaparte Gulf^20^. A previous global model^21^ shows an increasing trend of semi-diurnal tidal amplitude from present to the LGM in the northwestern Australia region. Previous paleo-tidal modeling did not consider changes in sea-level intermediate between the present and the LGM. In this study we model paleo-tidal amplitudes using intervals of 10 m sea-level change to explore in greater detail the change in tidal regime after isolation of the depression from the open ocean. Estimates of paleo-tidal amplitude are critically important when trying to accurately interpret indicators of relative sea-level change.

Here we present comprehensive global sea-level history from the full duration of MIS 2 from the Bonaparte Gulf (Fig. 1) through the application of glacial isostatic adjustment (GIA) modeling to a multi-proxy RSL record constructed from a transect of ten gravity cores. Over 100 radiocarbon measurements are performed to obtain good chronological control. A two-dimensional paleotidal model^22^ is used to evaluate the uncertainty introduced by Bonaparte’s relatively large tidal range^20^. The results document the evolution of large-glacial sea level with previously-unattained resolution, precision, and accuracy during MIS 2.

## Results

### Reconstruction of the sedimentary environment as a sea-level indicator

**Figure 2:**
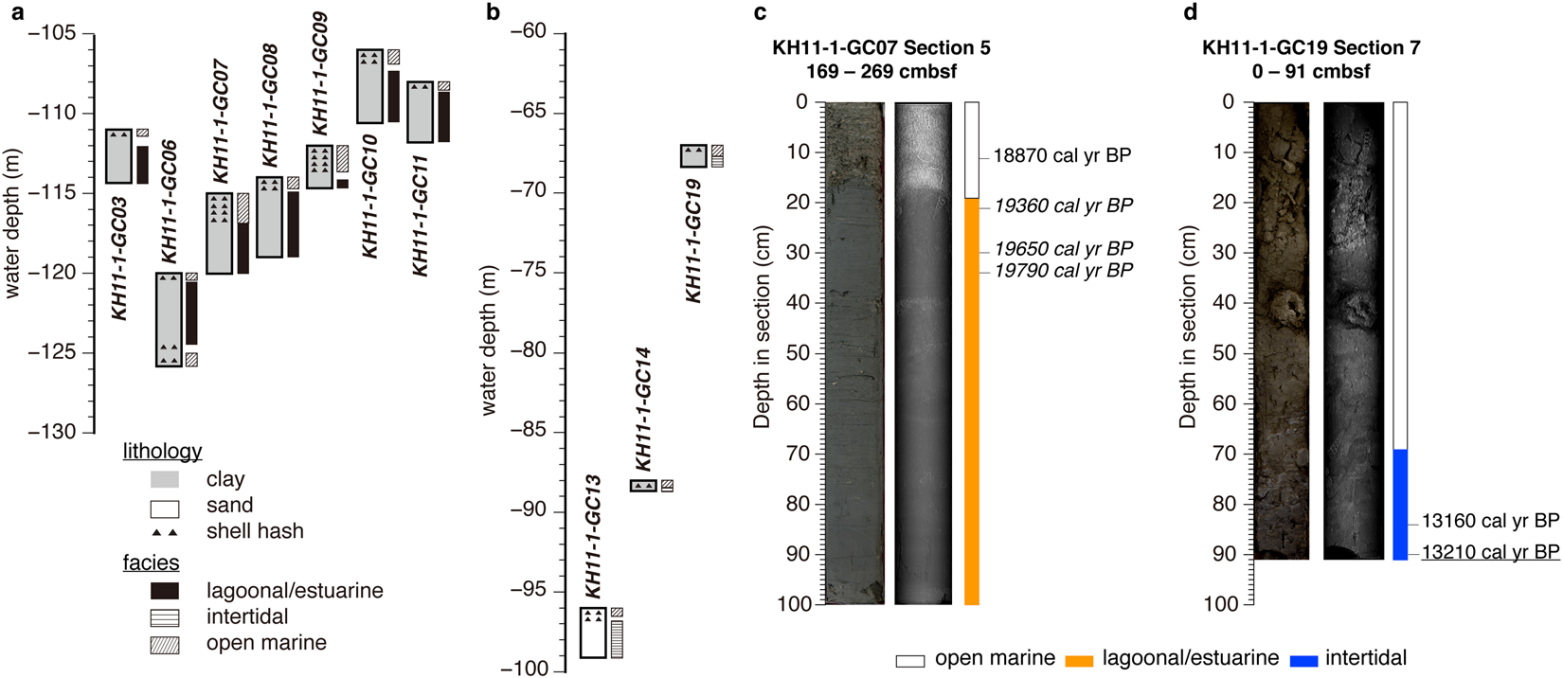
Lithological descriptions and facies of KH11-1 cores discussed in this study and core locations. (**a**) KH11-1 cores from −106 to −120 m water depth. (**b**) KH11-1 cores from −67 to −96 m water depth. The lagoonal/estuarine and intertidal facies are deposited below open marine facies that are indicative of high-energy tidal current activity during sea-level highstands (see Fig. 3). (**c**,**d**) Core photographs and X-ray radiographs of core sections with facies discussed in this study. Median calendar ages using MatCal^38^ are in the right column. Marine macrofossils (regular font), foraminifera (italic font), and plant fossils (underscored font).

The transect of cores were recovered from the Bonaparte Gulf, between a water depth of −120 and −67 m during cruise of KH-11-1 in 2011 (Figs 1, 2, and Supplementary Table S1). The range of water depth was selected to provide a continuous RSL record for MIS 2. Following previous work^23^, lagoonal/estuarine, intertidal, and open marine facies are documented in KH11-1 cores based on the abundance of particular benthic foraminifera, such as *Ammonia beccarii* (Methods and Supplementary Fig. S1), and terrestrial components (as indicated by C/N ratios and Ca/Ti ratios; Supplementary Figs S2 and S3). *A*. *beccarii* is a salt-marsh foraminifer useful for reconstructing Holocene sea-level changes^24^. The lagoonal/estuarine and intertidal facies are dominated by sedimentary components consistent with close proximity to the coastline, while the open marine facies consists of primarily marine materials indicated by low C/N ratios (<10) and high Ca/Ti ratios (Supplementary Figs S2 and S3). The lagoonal/estuarine facies is evident in sediment gravity cores KH11-1-GC10 and KH11-1-GC11 due to a dominantly terrestrial geochemical signature (as indicated by high C/N ratios (>10) and relatively low Ca/Ti ratios; Fig. 2a, Supplementary Fig. S2), as well as in KH11-1-GC03, GC06, GC07, GC08, and GC09 (Fig. 2b, Supplementary Fig. S2), which additionally contain a high abundance of *A*. *beccarii*. In KH11-1-GC14, GC19, and GC13 the intertidal facies is identified by abundant terrestrial organic matter with high C/N ratios (>20) and plant macrofossils in KH11-1-GC14 and GC19. The latter core also contains a prominent sand layer (Fig. 2a, Supplementary Fig. S3). The remaining intervals of each core contain evidence of strong bottom current activity with low C/N ratios (<10) and relatively high Ca/Ti ratios indicating an open marine setting with increased distance from the paleoshoreline.

### Age-depth model reconstruction

**Figure 3:**
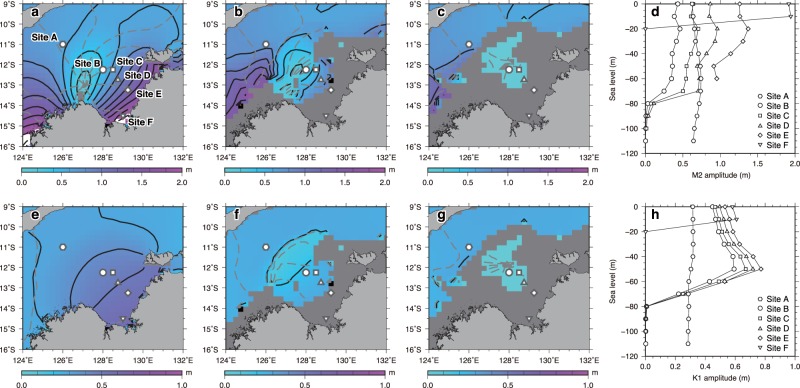
Changes in M2 (upper panels, (a–d)) and K1 (lower panels, (e–h)) tides with sea-level change. (**a**–**c**) Co-tidal charts of M2 tides for (**a**) present level, (**b**) sea level −70 m, and (**c**) −90 m. Color shades and black contours denote tidal amplitudes (c.i. 0.2 m) whereas gray dashed contours indicate co-phase lines (c.i. 30 degrees). (**d**) Variations in M2 amplitudes with sea-level change at six selected sites, indicated by symbols (**a**–**c**). Lower panels (e–h) are same as upper panels (a–d) but for K1 tides (c.i. 0.1 m for amplitudes and 30 degrees for phase). Note that the range of color scales adopted in (**a**–**c**) are different from that in (**e**–**g**) and that the amplitude range in (**d**) differs from that in (**h**).

In the lagoonal/estuarine facies, marine macrofossils are generally well preserved, associated with clay-rich sediments, and thus appear to be *in-situ* (Fig. 2c), which is consistent with tidal modeling results indicating low tidal energy during sea-level lowstands (Fig. 3). Conversely, construction of age models within the open marine facies was problematic due to a low number of well-preserved macrofossils and potential reworking of older materials due to high tidal energy during sea-level highstands (Fig. 3). Local radiocarbon reservoir age (ΔR) in the Bonaparte Gulf appears to be negligible because well-preserved marine and terrestrial macrofossils from the same stratigraphic level in KH11-1-GC19 exhibit consistent calendar ages following calibration by Marine13 and IntCal13^25^, respectively (Fig. 2d, Methods, and Supplementary Table S2).

### Paleo-tidal reconstruction

Modeled results for the K1 and M2 components, representing diurnal and semi-diurnal tides respectively, are greatly affected by glacial/interglacial-scale sea-level variability, while the K1, O1, M2, and S2 components are insensitive to such change (Supplementary Fig. S4). Lowering sea level from 0 m to −120 m results in a much lower tidal range for the K1 and M2 components (Fig. 3). In particular, tidal amplitude approaches zero once sea level drops below −90 m as large swaths of the bay are exposed. O1 and S2 tidal patterns are similar to those of K1 and M2 (Supplementary Fig. S4).

## Discussion

**Figure 4:**
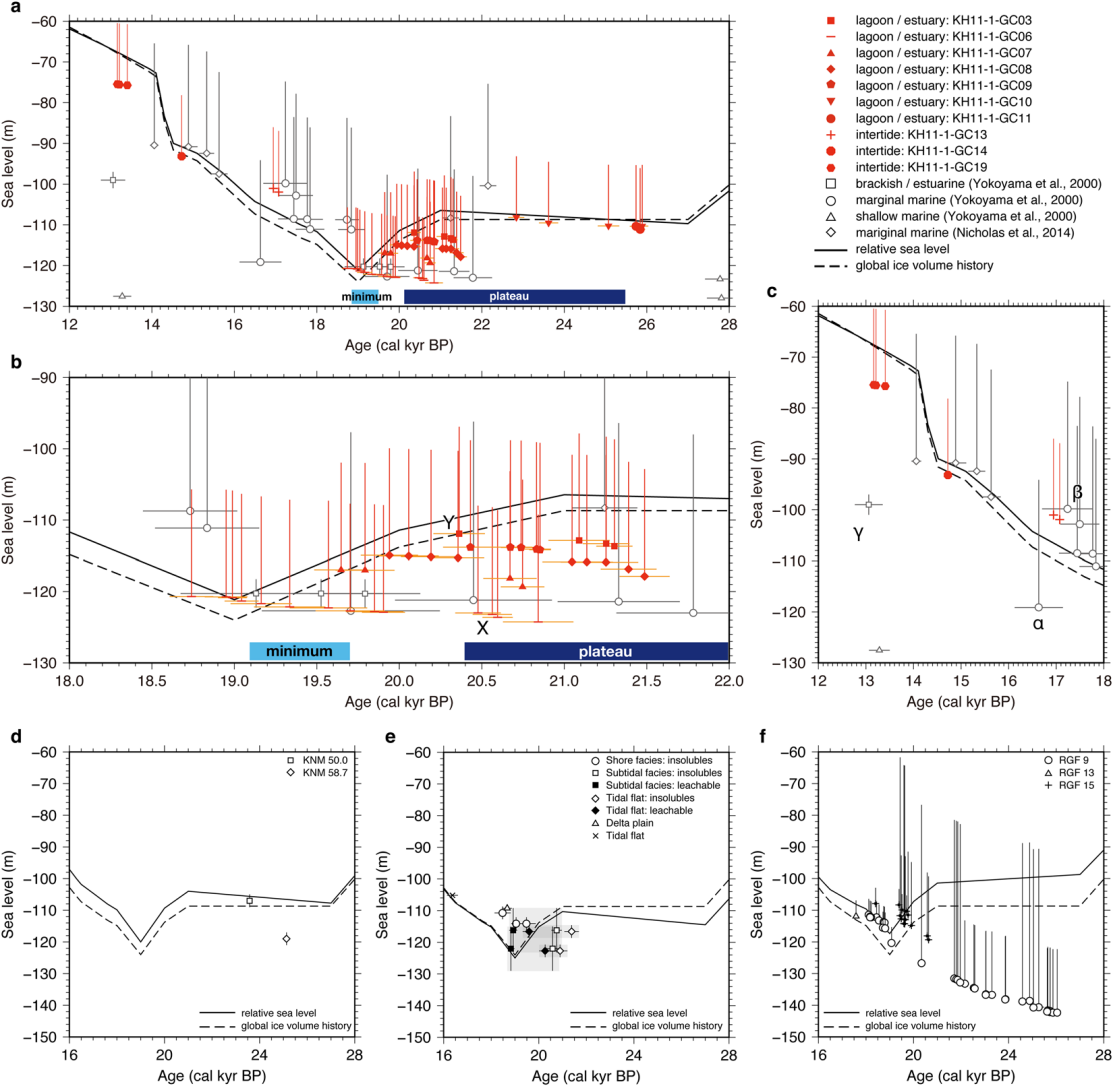
Illustration of sea-level change with observations and predictions for the Bonaparte Gulf and other MIS 2 sea-level records. Dashed lines correspond to ice volume equivalent sea level, and black lines to predictions at elastic lithosphere of 70 km, upper mantle viscosity of 4 × 10^20^ Pa s, and lower mantle viscosity of 5 × 10^22^ Pa s. (**a**) RSL data in the Bonaparte Gulf based on the depth-transect approach. The isostatic differences among cores are corrected to the sea-level equivalent value at KH11-1-GC06. Red symbols indicate data from this study. Whites correspond to data from previous work^13,14^. The light blue bar is the duration of sea-level minimum. The dark blue bar is the duration of sea-level plateau. The depth transect indicates that marginal marine facies^13,14^ occur within +25 m water depth and shallow marine^13,14^ facies occur over +25 m water depth. (**b**) Reconstruction limited 22 to 18 cal kyr BP. Symbols same as panel (a). The depth transect indicates that lagoonal/estuarine and intertidal facies occur within +15 m water depth using points from KH11-1-GC03 (point X) and GC06 (point Y) at ca. 20.5 cal kyr BP. (**c**) Reconstruction limited 12 to 18 cal kyr BP. The point α cannot be explained by a single sea-level curve deduce from point β if paleo-water depth uncertainty is +5 m, suggesting that the uncertainty of the marginal marine environment (inverted triangles) should be revised to +25 m. The brackish environment is observed at ca. 13 cal kyr BP (point γ)^13,14^. However, this core was not reevaluated in ref. ^14^. Other sea-level data from corals^6^ and sediments^8^ show the sea-level position above −80 m, suggesting that the paleo-water depth of this brackish facies is underestimated. (**d**) RSL reconstruction from the Huon Peninsula^10–12^. (**e**) RSL reconstruction from the Sunda Shelf^8,9^. Gray shades show radiocarbon ages of acid-insoluble and leachable organic matter in the same horizon. Uncertainties of age derive from the effect of old carbon^30^. (**f**) RSL reconstruction from Barbados^6,7^.

A depth transect of sediment cores is a powerful tool to overcome the limitation of an individual sea-level index point^13^. This approach assumes that two cores with different paleo-water depths at the same age may be explained by a single sea-level curve plus age uncertainty. Uncertainty in paleo-water depth may be constrained by evaluating the consistency between deep- and shallow-water indices (Supplementary Fig. S5). If a shallower sea-level point cannot be explained, water-depth uncertainty should be revised. We applied this method to our sea-level curve and re-evaluated previous work (Methods and Supplementary Fig. 4b,c). The depth transect indicates that lagoonal/estuarine and intertidal facies occur within +15 m water depth using points from KH11-1-GC03 (point X in Fig. 4b) and GC06 (point Y in Fig. 4b) at 20.5 cal kyr BP. This range is consistent with the present water depth of the estuarine environment in the Bonaparte Gulf (Supplementary Fig. S6). Here, a water-depth uncertainty of lagoonal/estuarine and intertidal facies of +15 m encompasses contemporaneous sea-level indices.

RSL can be deduced from the ages and depths of significant facies changes in this transect. The age-depth model of K11-1-GC10 indicates that sea level was above −110 m from 25.9 to 23.6 cal kyr BP (Fig. 4; modeled median age probability), which is consistent with KH11-1-GC11 indicating sea level above −110 m at 25.7 cal kyr BP. Little lithological (massively bedded clay matrix) and geochemical variation suggest only a minor variation of sea level at this time (Figs 2 and 4, Supplementary Fig. S2). While previous work^26^ reports a single datum at ca. 22 cal kyr BP with −94 m sea level, this may reflect reworking due to strong bottom currents during sea-level highstands. The age-depth model of KH11-1-GC03 shows that sea level was above −114 m from 21.3 to 20.4 cal kyr BP (Fig. 4b), which is consistent with the lagoonal/estuarine facies observed in KH11-1-GC06. The lagoonal/intertidal facies in KH11-1-GC03, GC10 and, GC11 indicate a sea-level plateau from 25.9 to 20.4 cal kyr BP, which is shallower than previously reported MIS 2 sea level^27^ (Supplementary Fig. S7). RSL above −117 m during 20.8 to 19.7 cal kyr BP is indicated by GC07 and consistent with sea level reconstructed from KH11-1-GC03, GC06, GC08 and GC09 (Fig. 4b). Previous work in the Bonaparte Gulf^13,14^ reports a brackish environment at −120 ± 2 m sea level during 19.8 to 19.1 cal kyr BP in core RS176-GC5 (Fig. 1). This agrees with the end of lagoonal/estuarine facies in KH11-1-GC07 (2σ age range). A rapid sea level rise^13,14^ indicates that the LGM sea-level minimum terminated at 19.1 cal kyr BP, corresponding to the demise of lagoonal/estuarine facies in KH11-1-GC06. Lagoonal/estuarine facies in KH11-1-GC06 and GC07, combined with the brackish environment in RS176-GC5^13,14^, support the interpretation that the LGM sea-level minimum in the Bonaparte Gulf occurred from 19.7 to 19.1 cal kyr BP (modeled median probability). Sea level then rose to above −98 m by ca. 17 cal kyr BP as indicated by the presence of an intertidal facies in KH11-1-GC13 (Fig. 4a,c). The intertidal facies observed in KH11-1-GC14 and GC19 further indicate a rapid sea-level rise at ca. 14 cal kyr BP, corresponding to Meltwater Pulse 1A interval^28^.

Our constraints on paleo-tidal amplitude indicate that tidal amplitude is reduced below −70 m sea level due to a shift of an amphidromic area (i.e., where tidal amplitudes take minimum values) and the changes in the basin configuration of the Bonaparte Gulf (Fig. 3). The Bonaparte Gulf was largely isolated from the open ocean due to lowered sea level with restricted connections between exposed carbonate platforms^16,17^ (Fig. 1). The location of submerged platforms could also be identified in the −70 m model results because they roughly coincide with a band of K1 amplitude minimum (Fig. 3) or an offshore limit of M2 amplitudes lower than 0.5 m (Fig. 3). The semi-enclosed environment during sea-level lowstands below −90 m could enhance deposition due to the decreased tidal influences in the Bonaparte Gulf and lack of bottom current scouring, suggesting that the environment is suitable to reconstruct RSL change during the exposure of carbonate platforms and terraces.

The 500-km width of the Bonaparte Gulf continental shelf causes a sea-level gradient across this shelf to appear due to a local difference in isostatic effect^13^ and bathymetry (Supplementary Fig. S8), which can be calculated using a GIA model. The facies-based observational RSL records described above can then be corrected to sea-level equivalent values at KH11-1-GC06 (Fig. 4) to consider the offset of GIA predictions among sites^29,30^. This global ice volume history model consistently explains observational RSL, indicating that a sea-level plateau occurred from 25.9 to 20.4 cal kyr BP, prior to the LGM (19.7 to 19.1 cal kyr BP; Fig. 4, Supplementary Fig. S8).

Considering the calculated regional isostatic effect, the Bonaparte Gulf RSL can be compared with other RSL records. Observational RSL records during MIS 2 and 3 using uplifted terraces in the Huon Peninsula^10–12^ are consistent with predicted RSL of this study’s ice model (Fig. 4d), supporting the previous-suggested uplift rate of the Huon Peninsula. A MIS-2 RSL record was previously created through sedimentary environmental reconstruction and radiocarbon dating of near-shore cores on the Sunda Shelf^8,9^ (Fig. 4e). Because the radiocarbon dating was performed on organic matter with leachable and insoluble components, it exhibits ca. 1,000 years difference within the same horizon, likely due to the effect of old carbon^31^. The Sunda Shelf record is also consistent with our global ice volume history within age uncertainties derived from the effect of old carbon^31^. Additionally, MIS 2 RSL has been reconstructed through precise U-series dating of Barbados corals (Fig. 4f)^6,7^. However, inconsistent results prior to 19 cal kyr BP suggest differential uplift rates between sites, perhaps caused by faulting^32^. There is also large uncertainty in the growth positions (20–50 m) of particular species (*Montastrea annularis*, *P*. *asteroids*, and *Diploria*)^7^.

Rapid ice-sheet growth of approximately 10 m ice-volume equivalent sea-level between the sea-level plateau prior to the LGM and the time of minimum RSL is observed in our RSL records and the GIA modeling (Fig. 4a,b). This would primarily be ice in the Northern hemisphere because the maximum volume added to the Antarctic ice sheet during the LGM is reported to be less than 10 m^33^. The extremely short duration of the global ice volume maximum, revealed by our results, has implications for the usage of LGM boundary conditions for modeling MIS 2 climate since continental ice sheets likely never reached isostatic equilibrium. This could lead to overestimating the maximum volume of each continental ice sheet, which in total may be closer to that inferred from far-field sea-level records^34^. However, additional MIS 2 records are needed to more fully explore these implications. In particular, near-field sites with highly-resolved age dating are required to understand the discrepancy between reconstructed global ice volume and the total estimated from individual ice-sheets by near-field dataset^34^. We conclude that a sea-level plateau occurred from 25.9 to 20.4 cal kyr BP with a subsequent 10 m sea-level fall in ~1,000 years, suggesting that continental ice sheets during MIS 2 were less stable than previously believed^27^ (Supplementary Fig. S7). Future work reconstructing paleoclimate during glacial periods should consider the instability in sea level and ice volume revealed by this research.

## Methods

Geochemical analyses were performed on sediments to determine the relative contribution of marine and terrestrial components to detect changes in the distance from the coastline. C/N ratios and δ^13^C were measured at a 4 to 10-cm interval using EA-IRMS (Elemental Analysis-Isotope Ratio Mass Spectrometry; Flash EA 1112 and Delta plus Advantage) at the Center for Advanced Marine Core Research (CAMCR) in Kochi University^15,17^. X-ray fluorescence (XRF) core scanning was conducted at a 1-cm interval using TATSCAN-F2 at the CAMCR^35^.

Approximately 100 radiocarbon measurements were performed on marine macrofossils, benthic foraminifera, and terrestrial macrofossils (Supplementary Table S2). Marine macrofossils and foraminifera were cleaned using an ultrasonic cleaner and then etched by 10 M HCl to remove secondary and/or contaminating carbonate materials, which is often attached to the surface of samples. Graphitization was performed following previously reported methods^36^. The analyses were performed at The University of Tokyo using a Tandem Accelerator at the Micro Analysis Laboratory and a Single Stage AMS at Atmosphere and Ocean Research Institute^37^.

Radiocarbon ages were calibrated to calendar ages using MatCal^38^ with Marine13 for marine macrofossils and foraminifera and IntCal13 for terrestrial macrofossils^25^. The local reservoir correction is not known for the Bonaparte Gulf but is likely very small^39,40^. Therefore we did not apply a local reservoir (ΔR) correction. Age modeling for cores was performed using 75 of the radiocarbon dates in a 2000-iteration Monte Carlo simulation adapted from ref.^41^, where one probability-weighted age per iteration was selected from the 95.4% range of each calibrated age probability density function (PDF). Over 20 radiocarbon dates are excluded from age modeling due to the reversed age-depth relation. Sampling was repeated up to 1000 times if an age reversal occurred. The calendar year age at each stratigraphic level is the median age of all non-reversing iterations, and uncertainty is reported as the 2.3 and 97.7 percentiles (95.4% range).

Paleotidal variations were reconstructed using a two-dimensional model^22^ covering longitude 115°E–143°E and latitude 8.5°S–22°S with the resolution of 1/4 degrees. The simulation was conducted by lowering the sea level uniformly every 10 m from 0 m to −120 m and neglected the impact of isostatic deformation on the seafloor because the Bonaparte Gulf is tectonically stable and far from the former ice sheet^13–15,42^. Tides were forced by specifying tidal elevations along open boundaries compiled from harmonic constants of diurnal (K1 and O1) and semi-diurnal (M2 and S2) constituents of a modern global tidal model (TPXO8-atlas)^43^. The M2 constituent is the largest semi-diurnal tidal component and K1 is the largest diurnal component, indicating that the variations of these two constituents reflect the general tidal evolution in the Bonaparte Gulf. We applied the present-day values at the model grid boundaries because global paleotidal models show that tidal elevations along the offshore boundaries did not significantly fluctuate during MIS 2^21,44^ and also because modern ocean tides are well-constrained by satellite altimetry, which is not available for the geological past.

A reconstruction of the paleoecological environment of Bonaparte Gulf from vibracore data was presented by ref.^23^. In this previous study, six environments were identified based on biological assemblage: intertidal, lagoonal, estuarine, strandlines, shelf, and riverine with occurrence of *A*. *beccarii* indicating lagoonal and estuarine environments. Studies using sediments from Europe, Australia, and East China Sea also support lagoonal and estuarine habitats for *A*. *beccarii*^45–47^. We conducted a qualitative analysis of *A*. *beccarii* abundance, classifying it as barren, low, and high (Supplementary Fig. S1).

The previous work^13,42^ assigned an uncertainty of +5 m to a marginal marine facies based on an assemblage indicative of normal sea-water salinity^14^. However, data at ca. 16.5 cal kyr BP (Fig. 4c; point α) cannot be explained by a single sea-level curve deduced from point β (Fig. 4c), suggesting the need for revision. Studies on RSL during the deglaciation agree with a sea-level curve deduced from point β, requiring adjusting depth uncertainty for this facies to +25 m. This adjustment is also supported by an apparent mismatch in the age and magnitude of the post-LGM sea level rise^29^ indicated by other sea-level records such as Barbados^6,7^ and Sunda Shelf^8,9^.

The GIA model is based on the below sea-level equation^48,49^. RSL at site ${\rm{\phi }}$ and time t (${{\rm{\Delta }}{\rm{\zeta }}}_{{\rm{rsl}}}({\rm{\phi }},{\rm{t}})$) are expressed as follows:

1 $${{\rm{\Delta }}{\rm{\zeta }}}_{{\rm{rsl}}}({\rm{\phi }},{\rm{t}})={{\rm{\Delta }}{\rm{\zeta }}}_{{\rm{esl}}}({\rm{t}})+{{\rm{\delta }}{\rm{\zeta }}}_{{\rm{water}}}^{{\rm{iso}}}({\rm{\phi }},{\rm{t}})+{{\rm{\delta }}{\rm{\zeta }}}_{{\rm{ice}}}^{{\rm{iso}}}({\rm{\phi }},{\rm{t}})$$

where ${{\rm{\Delta }}{\rm{\zeta }}}_{{\rm{esl}}}({\rm{t}})$ is ice volume equivalent sea level (ESL) and ${{\rm{\delta }}{\rm{\zeta }}}_{{\rm{ice}}}^{{\rm{iso}}}({\rm{\phi }},{\rm{t}})$ and ${{\rm{\delta }}{\rm{\zeta }}}_{{\rm{water}}}^{{\rm{iso}}}({\rm{\phi }},{\rm{t}})$ is the glacio- and hydro- isostatic contributions, respectively. Variations in ${{\rm{\delta }}{\rm{\zeta }}}_{{\rm{ice}}}^{{\rm{iso}}}({\rm{\phi }},{\rm{t}})$ and ${{\rm{\delta }}{\rm{\zeta }}}_{{\rm{water}}}^{{\rm{iso}}}({\rm{\phi }},{\rm{t}})$ are controlled by the earth model, which is described by the elastic thickness of the lithosphere (H), upper-mantle viscosity (η_um_), and lower mantle viscosity (η_lm_). The parameters of the earth model are based on the PREM^50^ for the density and elastic constants. The adopted earth parameters in this study are based on the previous work^27,31^. We set H, η_um_, and η_lm_ to 70 km, (1 − 9) × 10^20^ Pa s, and (0.5 − 0.9, 1.0 − 10) × 10^22^ Pa s, respectively.

The ANU model cannot explain the sea-level plateau from 25.9 to 20.4 cal kyr BP within the range of predictions derived by the earth model (Supplementary Fig. S7c). ESL in this study’s ice model is based on the ANU model and multiplied with different constant factors for each time-step. The ratio of each continental ice-sheet volume in this study’s ice model is the same as the ANU model.

## Supplementary information

Supplementary Information

## References

[1] Lambeck K, Esat TM, Potter EK (2002). Links between climate and sea levels for the past three million years. Nature.

[2] Abe-ouchi A (2013). Insolation-driven 100,000-year glacial cycles and hysteresis of ice-sheet volume. Nature.

[3] Lisiecki LE, Raymo ME (2005). A Pliocene-Pleistocene stack of 57 globally distributed benthic δ^18^O records. Paleoceanography.

[4] Clark PU (2009). The Last Glacial Maximum. Science.

[5] Yokoyama Y, Esat TM (2011). Global climate and sea level: Enduring variability and rapid fluctuations over the past 150,000 years. Oceanography.

[6] Fairbanks RG (1989). A 17,000-year glacio-eustatic sea level record: influence of glacial melting rates on the Younger Dryas event and deep-ocean circulation. Nature.

[7] Peltier WR, Fairbanks RG (2006). Global glacial ice volume and Last Glacial Maximum duration from an extended Barbados sea level record. Quaternary Science Reviews.

[8] Hanebuth T, Stattegger K, Grootes PM (2000). Rapid Flooding of the Sunda Shelf: A Late-Glacial Sea-Level Record. Science.

[9] Hanebuth T, Stattegger K, Bojanowski A (2009). Termination of the Last Glacial Maximum sea-level lowstand: The Sunda-Shelf data revisited. Glob. Planet. Change.

[10] Yokoyama Y, Esat TM, Lambeck K (2001). Coupled climate and sea-level changes deduced from Huon Peninsula coral terraces of the last ice age. Earth. Planet. Sci. Lett..

[11] Yokoyama Y, Esat TM, Lambeck K (2001). Last glacial sea-level change deduced from uplifted coral terraces of Huon Peninsula, Papua New Guinea. Quaternary International.

[12] Cutler KB (2003). Rapid sea-level fall and deep-ocean temperature change since the last interglacial period. Earth. Planet. Sci. Lett..

[13] Yokoyama Y, Lambeck K, De Deckker P, Johnston P, Fifield LK (2000). Timing of the Last Glacial Maximum from observed sea-level minima. Nature.

[14] De Deckker P, Yokoyama Y (2009). Micropalaeontological evidence for Late Quaternary sea-level changes in Bonaparte Gulf, Australia. Glob. Planet. Change.

[15] Ishiwa T (2016). Reappraisal of sea-level lowstand during the Last Glacial Maximum observed in the Bonaparte Gulf sediments, northwestern Australia. Quaternary International.

[16] Bourget J, Ainsworth RB, Backe G, Keep M (2012). Tectonic evolution of the northern Bonaparte Basin: impact on continental shelf architecture and sediment distribution during the Pleistocene. Australian Journal of Earth Sciences.

[17] Ishiwa T, Yokoyama Y, Miyairi Y, Ikehara M, Obrochta S (2016). Sedimentary environmental change induced from late Quaternary sea-level change in the Bonaparte Gulf, northwestern Australia. Geoscience Letters.

[18] Shennan I, Milne G (2003). Sea-level observations around the Last Glacial Maximum from the Bonaparte Gulf, NW Australia. Quaternary Science Reviews.

[19] Yokoyama Y, De Deckker P, Lambeck K (2003). Reply to Sea-level observations around the Last Glacial Maximum from the Bonaparte Gulf, NW Australia by I. Shennan and G. Milne. Quaternary Science Reviews.

[20] Anderson, T. J. *et al*. Seabed Environments of the Eastern Joseph Bonaparte Gulf, Northern Australia: GA0325/Sol5117-Post- survey Report. *Geoscience Australia Record* 2011.08 (2011).

[21] Griffiths SD, Peltier WR (2009). Modeling of polar ocean tides at the Last Glacial Maximum: Amplification sensitivity, and climatological implications. J. Clim..

[22] Uehara K, Scourse JD, Horsburgh KJ, Lambeck K, Purcell A (2006). Tidal evolution of the northwest European shelf seas from the Last Glacial Maximum to the present. Journal of Geophysical Research: Oceans.

[23] Clarke JDA (2001). Post-glacial from the inner part of Southwest Joseph Bonaparte Gulf. Australian Journal of Earth Sciences.

[24] Horton BP (2007). Reconstructing Holocene Sea-Level Change for the Central Great Barrier Reef (Australia) Using Subtidal Foraminifera. Journal of Foraminiferal Research.

[25] Reimer PJ (2013). Intcal13 and marine13 radiocarbon age calibration curves 0 –50,000 years cal bp. Radiocarbon.

[26] Nicholas WA (2014). Pockmark development in the Petrel Sub-basin, Timor Sea, Northern Australia: Seabed habitat mapping in support of CO2 storage assessments. Continental Shelf Research.

[27] Lambeck K, Rouby H, Purcell A, Sun Y, Sambridge M (2014). Sea level and global ice volumes from the Last Glacial Maximum to the Holocene. Proceedings of the National Academy of Sciences.

[28] Deschamps P (2012). Ice-sheet collapse and sea-level rise at the Bølling warming 14,600 years ago. Nature.

[29] Lambeck K, Yokoyama Y, Purcell T (2002). Into and out of the Last Glacial Maximum: sea-level change during Oxygen Isotope Stages 3 and 2. Quaternary Science Reviews.

[30] Liu J, Milne GA, Kopp RE, Clark PU, Shennan I (2016). Sea-level constraints on the amplitude and source distribution of Meltwater Pulse 1A. Nature Geoscience.

[31] Nakada M, Okuno J, Yokoyama Y (2016). Total meltwater volume since the Last Glacial Maximum and viscosity structure of Earth’s mantle inferred from relative sea level changes at Barbados and Bonaparte Gulf and GIA-induced J2. Geophysical Journal International.

[32] Bard E, Hamelin B, Delanghe-Sabatier D (2010). Deglacial meltwater pulse 1B and Younger Dryas sea levels revisited with boreholes at Tahiti. Science.

[33] Whitehouse PL, Bentley MJ, Le Brocq AM (2013). A deglacial model for Antarctica: Geological constraints and glaciological modelling as a basis for a new model of Antarctic glacial isostatic adjustment. Quaternary Science Reviews.

[34] Clark PU, Tarasov L (2014). Closing the sea level budget at the Last Glacial Maximum. Proceedings of the National Academy of Sciences.

[35] Sakamoto T (2006). Non-Destructive X-Ray Fluorescence (XRF) Core-Imaging Scanner, TATSCAN-F2. Scientific Drilling.

[36] Yokoyama Y, Miyairi Y, Matsuzaki H, Tsunomori F (2007). Relation between acid dissolution time in the vacuum test tube and time required for graphitization for AMS target preparation. Nuclear Instruments and Methods in Physics.

[37] Yokoyama Y (2016). Widespread collapse of the Ross Ice Shelf during the late Holocene. Proceedings of the National Academy of Sciences.

[38] Lougheed BC, Obrochta SP (2016). MatCal: Open source Bayesian ^14^C age calibration in MatLab. Journal of Open Research Software.

[39] Bowman GM (1985). Oceanic reservoir correction for marine radiocarbon dates from northwestern Australia. Australian Archaeology.

[40] O’Connor S, Ulm S, Fallon SJ, Barham A, Loch I (2010). Pre-bomb marine reservoir variability in the Kimberley Region, Western Australia. Radiocarbon.

[41] Obrochta, S. P. *et al*. The undatables: Quantifying uncertainty in a highly expanded Late Glacial - Holocene sediment sequence recovered from the deepest Baltic Sea basin - IODP Site M0063. *Geochemistry*, *Geophysics*, *Geosystems* **18** (2017).

[42] Yokoyama Y, De Deckker P, Lambeck K, Johnston P, Fifield LK (2001). Sea-level at the Last Glacial Maximum: evidence from northwestern Australia to constrain ice volumes for oxygen isotope stage 2. Palaeogeogr. Palaeoclimatol. Palaeoecol..

[43] Egbert GD, Erofeeva SY (2002). Efficient inverse modeling of barotropic ocean tides. Journal of Atmospheric and Oceanic Technology.

[44] Egbert GD, Ray RD, Bills BG (2004). Numerical modeling of the global semidiurnal tide in the present day and in the last glacial maximum. J. Geophys. Res..

[45] Yassini I, Jones BG (1985). Recent Foraminifera and Ostracoda from Estuarine and Shelf Environments on the South-eastern Coast of Australia.

[46] Li C, Chen Q, Zhang J, Yang S, Fan D (2000). Stratigraphy and paleoenvironmental changes in the Yangtze Delta during the Late Quaternary. Journal of Asian Earth Sciences.

[47] Horton BP, Edwards RJ (2006). Quantifying Holocene sea level change using intertidal foraminifera: lessons from the British Isles. Cushman Foundation for Foraminiferal Research, Special Publication.

[48] Farrell WE, Clark JA (1976). On Postglacial Sea Level. Geophysical Journal of the Royal Astronomical Society.

[49] Nakada M, Lambeck K (1987). Glacial rebound and relative sea-level variations: a new appraisal. Geophys. J. R. Astron. Soc..

[50] Dziewonski AM, Anderson DL (1981). Preliminary reference Earth model. Physics of the Earth and Planetary Interiors.

[51] Whiteway, T. Australian Bathymetry and Topography Grid, June 2009. Scale 1:5000000. *Geoscience Australia Canberra* (2009).

[52] Wessel P, Smith WHF, Scharroo R, Luis JF, Wobbe F (2013). Generic Mapping Tools: Improved version released. EOS Trans. AGU.

