# Supplementary material for: A sea-level plateau preceding the Marine Isotope Stage 2 minima revealed by Australian sediments
Source: Sci Rep. 2019 May 10;9:6449. doi: 10.1038/s41598-019-42573-4 (PMC6509117; doi:10.1038/s41598-019-42573-4)
Supplement: Supplementary file 1 — Supplementary Information [file 41598_2019_42573_MOESM1_ESM.pdf]

Supplementary Information

A sea-level plateau preceding the Marine Isotope Stage 2 minima revealed by Australian sediments

Ishiwa et al.

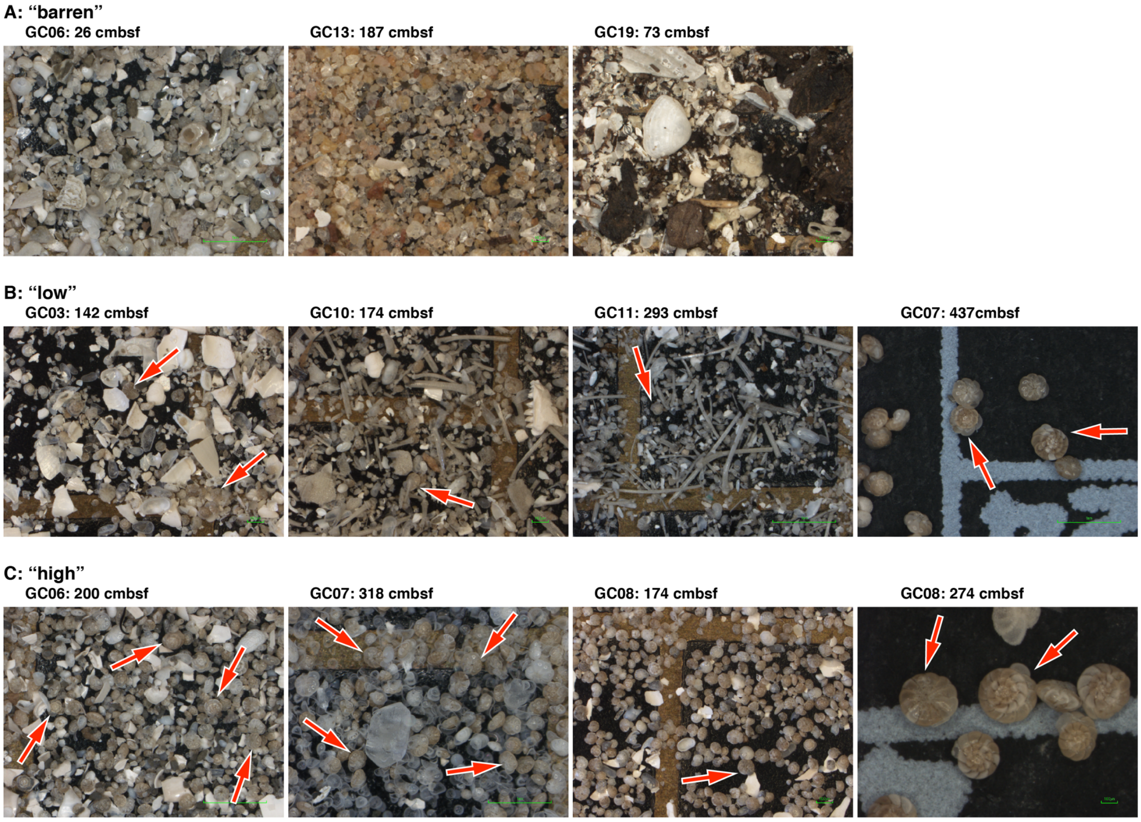

Figure S1 | Photos of sediments washed with 63 µm sieves. Abundance of *A. beccarii*, **(a)** barren, **(b)** low, and **(c)** high. Red arrows are examples of *A .beccarii*.

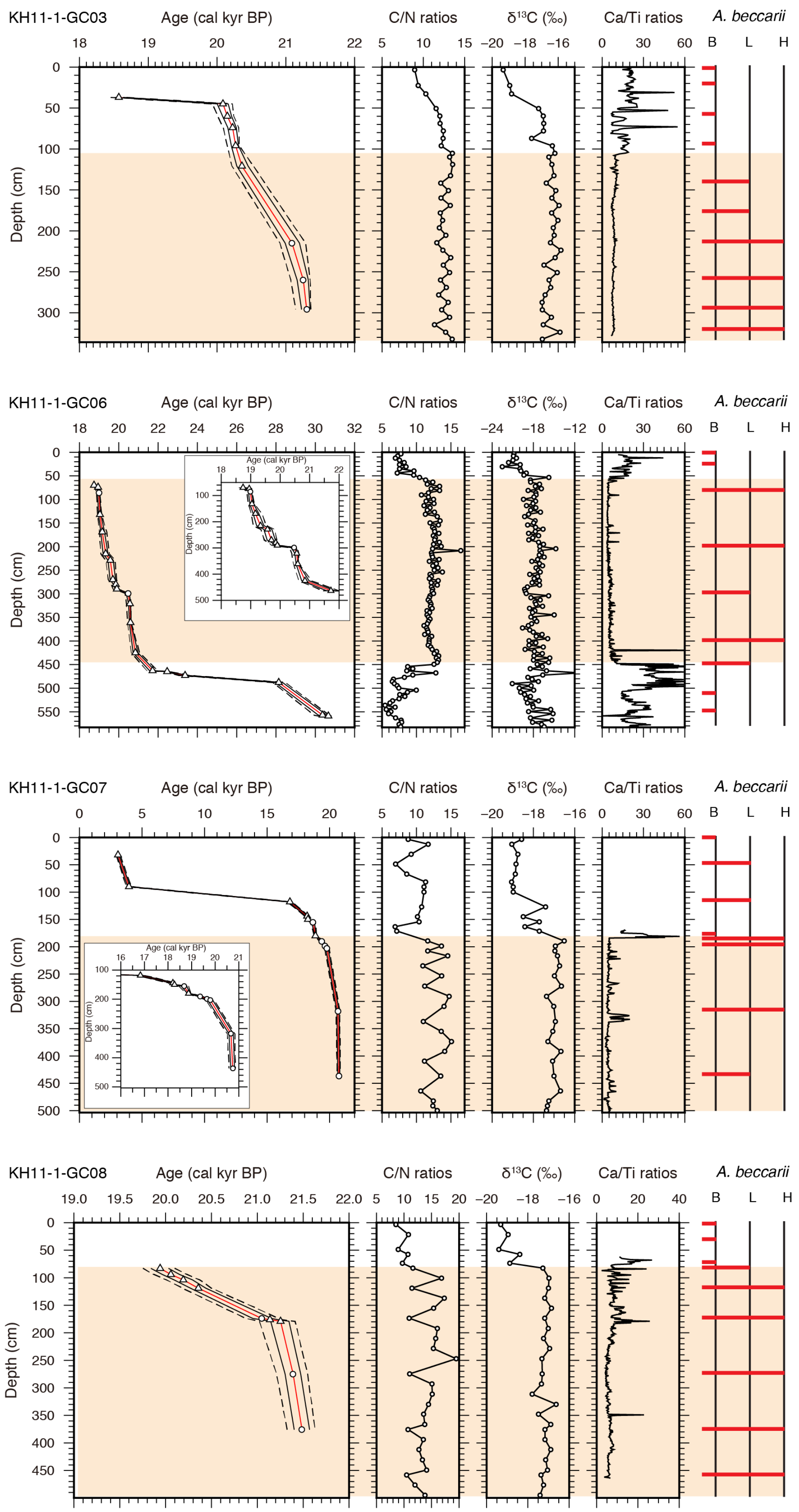
KH11-1-GC03
Age (cal kyr BP)
C/N ratios
δ13C (‰)
Ca/Ti ratios
A. beccarii
B L H
Depth (cm)
KH11-1-GC06
Age (cal kyr BP)
C/N ratios
δ13C (‰)
Ca/Ti ratios
A. beccarii
Depth (cm)
KH11-1-GC07
Age (cal kyr BP)
C/N ratios
δ13C (‰)
Ca/Ti ratios
A. beccarii
Depth (cm)
KH11-1-GC08
Age (cal kyr BP)
C/N ratios
δ13C (‰)
Ca/Ti ratios
A. beccarii
Depth (cm)

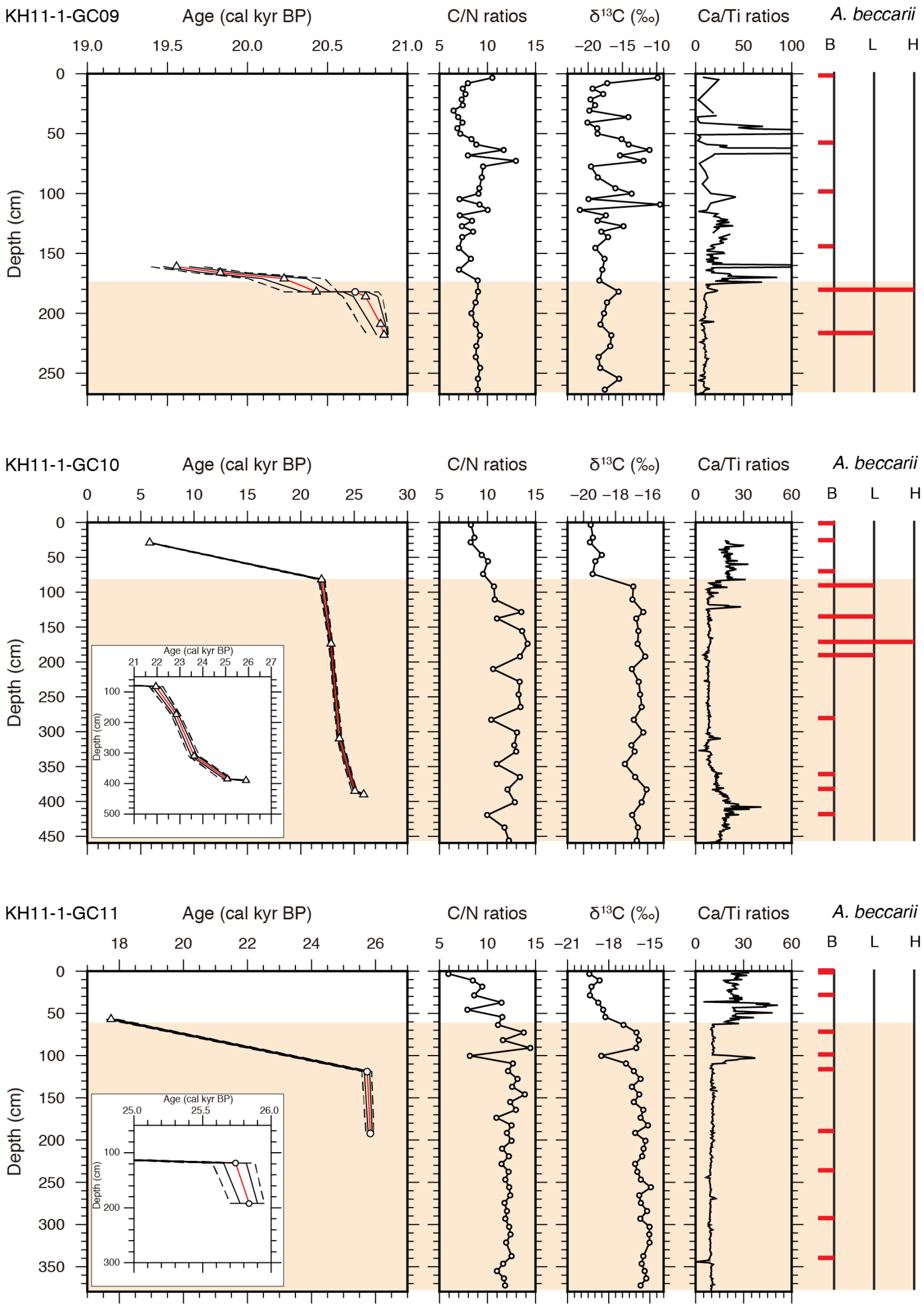

Figure S2 | Age-depth models, C/N ratios, δ^{13}C, and Ca/Ti ratios of KH11-1-GC03, GC06, GC07, GC08, GC09, GC10 and GC11 with the abundance of *A. beccarii*. The lagoonal/estuarine facies (orange shade) are observed in these cores. In age-depth models, triangles correspond to macrofossils date and circles to foraminifera date. Dashed lines are 2 sigma probability age and solid lines are 1 sigma probability age, which are calculated by Matcal[1].

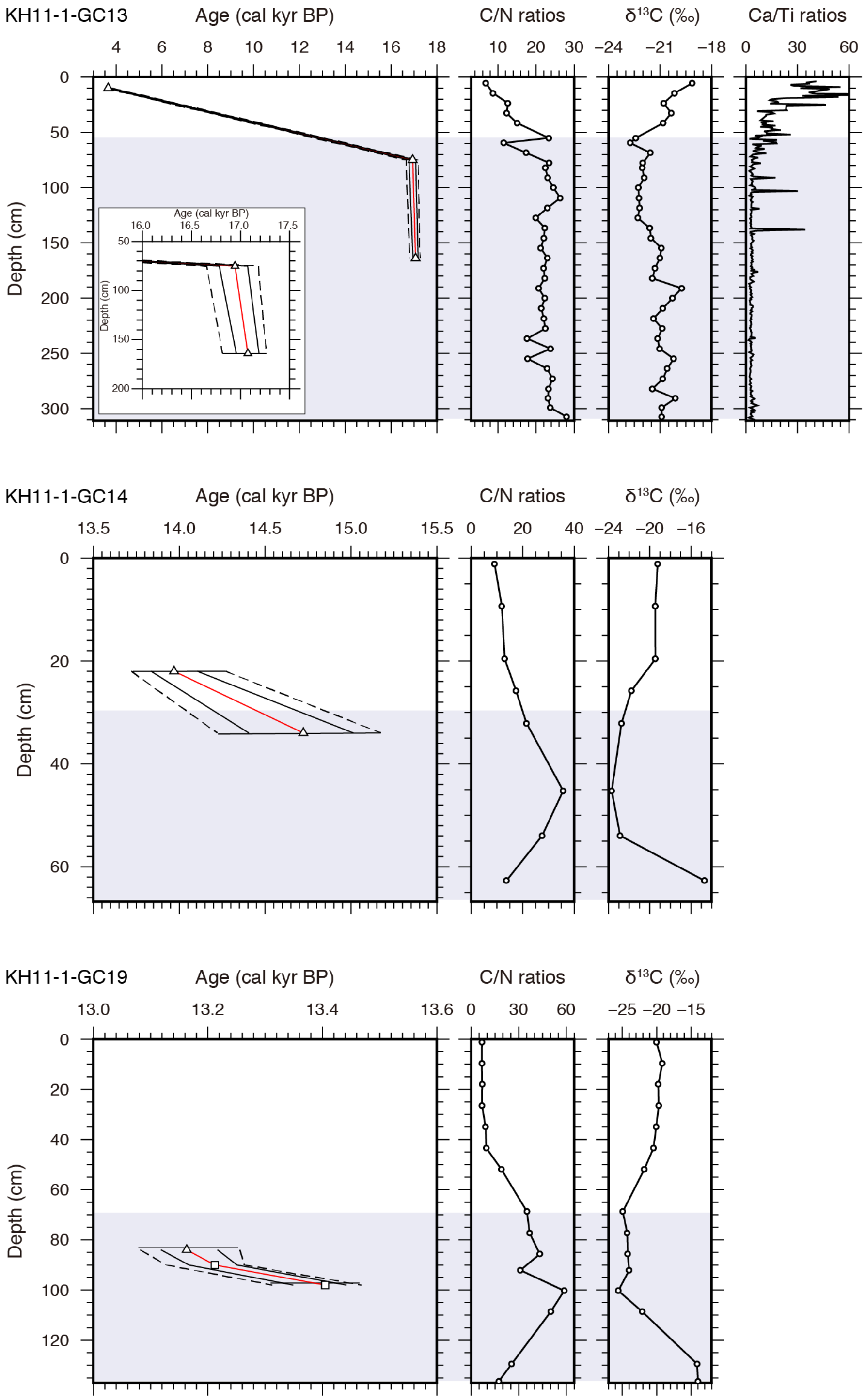

Figure S3 | Age-depth models, C/N ratios, δ13C, and Ca/Ti ratios of KH11-1-GC13, GC14, and GC19 with the abundance of *A. beccarii*. The intertidal facies (blue shade) are observed in these cores. In age-depth models, triangles correspond to macrofossils date, circles to foraminifera date, and squares to peat date. Dashed lines are 2 sigma probability age and solid lines are 1 sigma probability age, which are calculated by Matcal[1].

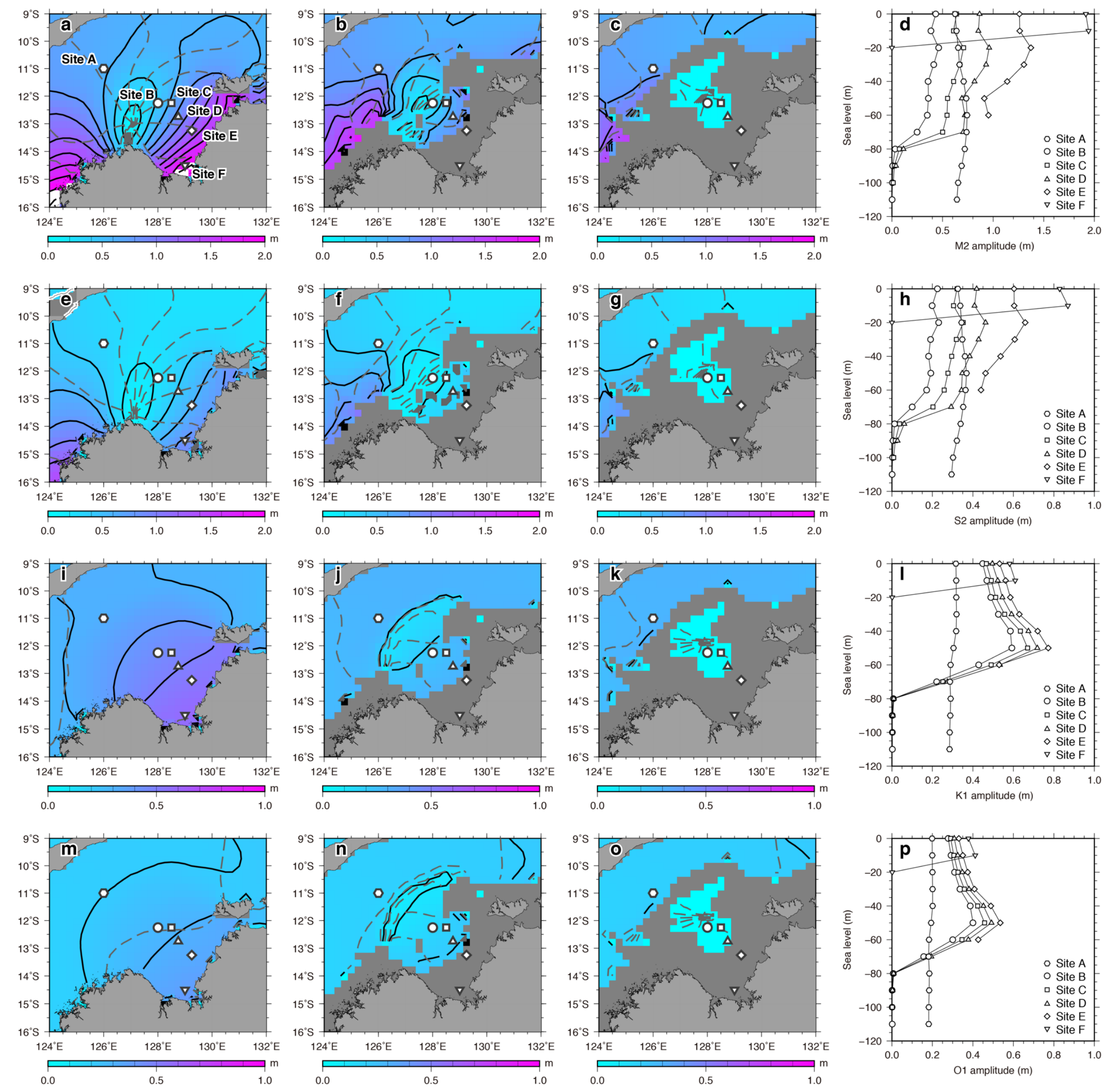

Figure S4 | Changes in M2, S2, K1, and O1 tidal constituents. (a-c) Co-tidal charts of M2 tides for **(a)** present level, **(b)** sea level -70 m, and **(c)** -90 m. Color shades and black contours denote tidal amplitudes whereas gray dashed contours indicate co-phase lines. **(d)** Variation of M2 tides with sea-level change at six selected sites, locations of which are indicated in **(a-c)** as symbols. Lower panels are same as **(a-d)** but for **(e-h)** S2 tides, **(i-l)** K1 tides, and **(m-p)** O1 tides. Contour intervals of tidal amplitudes are 0.2 m for semi-diurnal (M2 and S2) tides, 0.1 m for diurnal (K1 and O1) tides, and those of phases are 30 degrees for all tidal constituents.

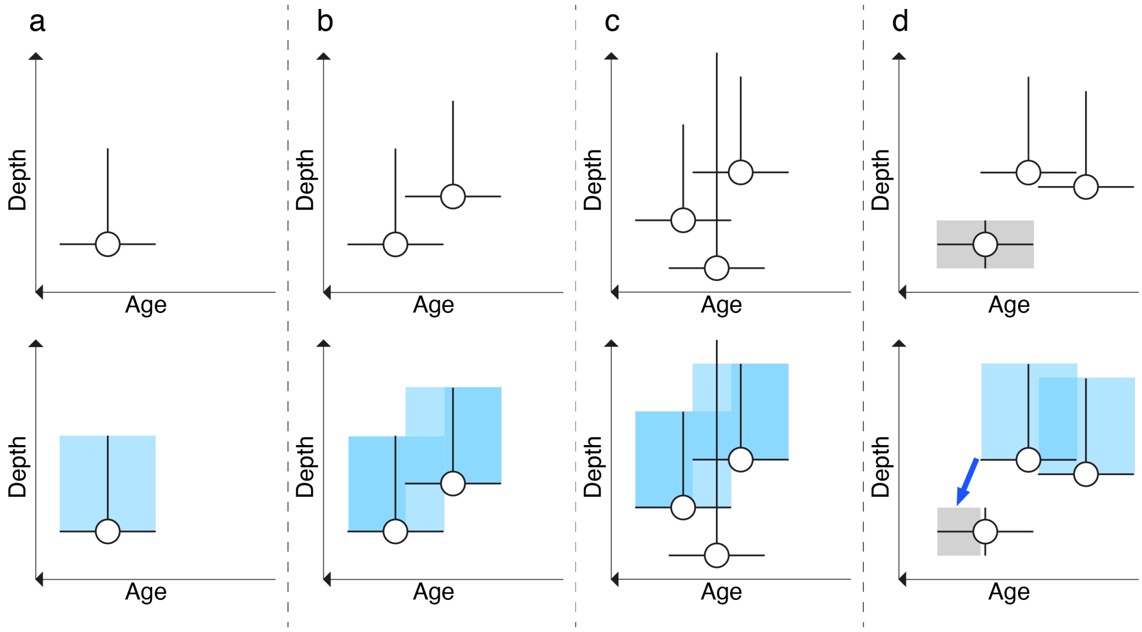

Figure S5 | Schematic model of paleo-water depth reconstruction. Dark blue and light grey shade are potential locations of sea level. **(a)**, **(b)**, and **(c)** Two cores with different depths at the same age should be explained with a single sea-level curve. If a shallower sea-level point is not explained, water-depth uncertainty should be revised. **(d)** Deeper core with the upper and lower limit and shallower core with the lower limit should be explained using a single sea-level curve within the age uncertainties of two cores.

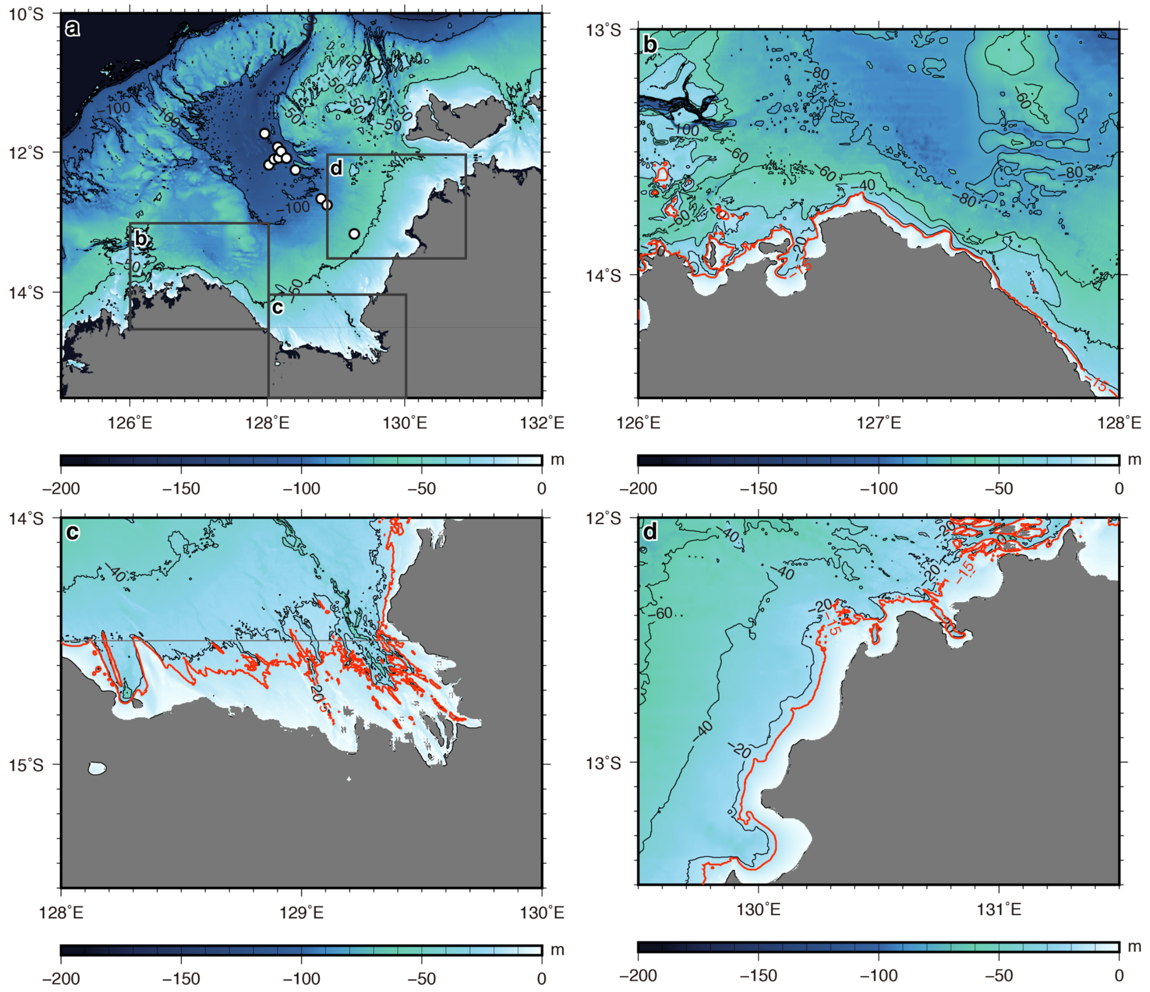

Figure S6 | Bathymetry of the Bonaparte Gulf. (a) A general view of the Bonaparte Gulf with 50-m contour. **(b)**, **(c)**, and **(d)** Extended figures of estuarine environment. Black lines are every 20-m contours and red lines are -15 m contour. Data from Geoscience Australia[2].

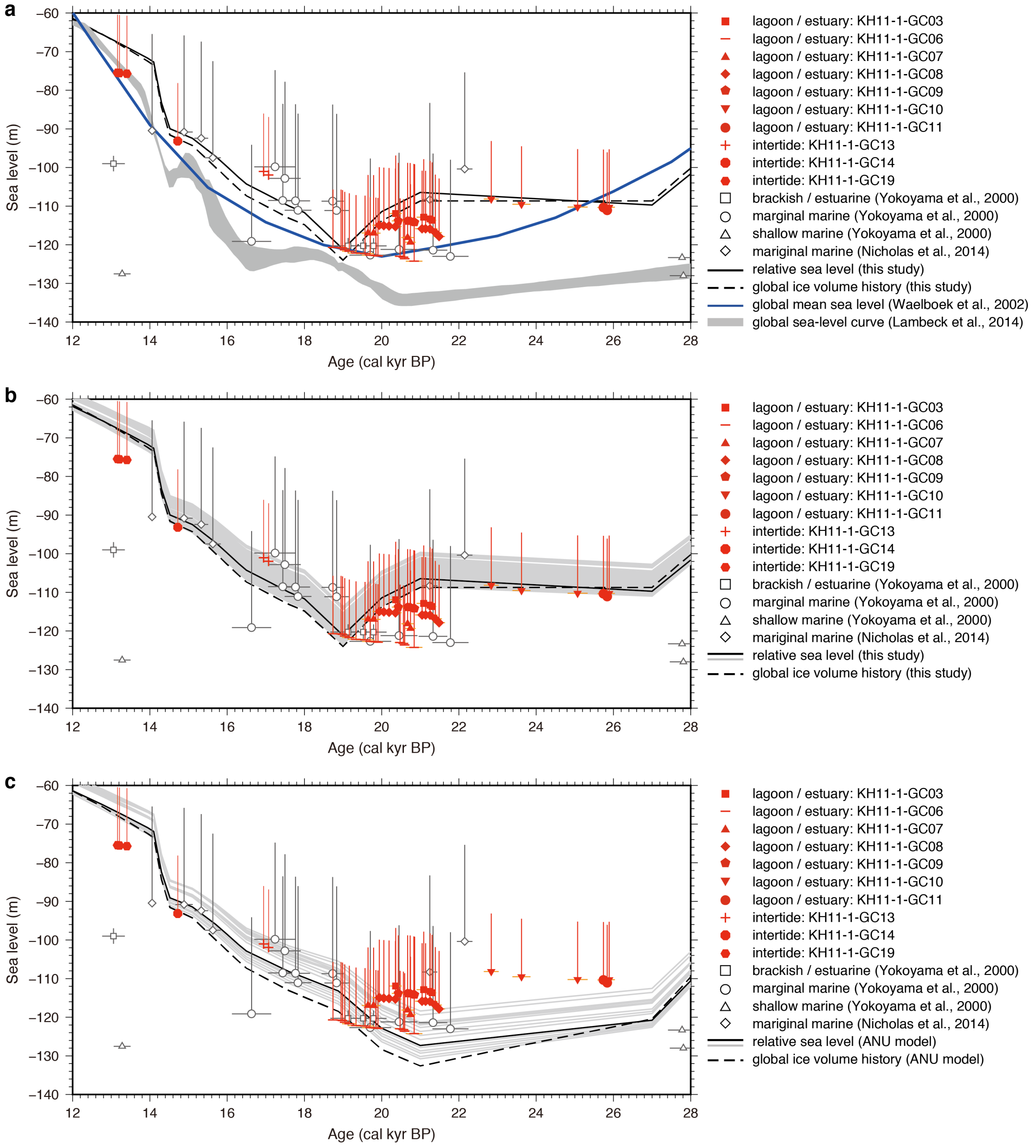

Figure S7 | Illustration of sea-level change in the Bonaparte Gulf with previous global sea-level curves. **(a)** RSL observations in the Bonaparte Gulf with GIA predictions using this study's ice model and other global sea-level history[5,6]. A black dashed line corresponds to ice volume equivalent sea level of this study's ice model, and a black solid line to RSL predictions at elastic lithosphere, upper mantle viscosity, and lower mantle viscosity to 70 km, 4×10^{20} Pa s, and 5×10^{22} Pa s. Gray shade is global sea-level curve from ref. 5. A blue line is global sea-level curve from ref. 6. **(b)** RSL observations in the Bonaparte Gulf with GIA predictions using this study's ice model. A black dashed line corresponds to ice volume equivalent sea level**.** Gray lines are GIA predictions at elastic lithosphere, upper mantle viscosity, and lower mantle viscosity to

70 km, $(1\text{–}9) \times 10^{20}$ Pa s, and $(0.5 - 0.9, 1.0\text{–}10) \times 10^{22}$ Pa s. A black line is GIA prediction at elastic lithosphere, upper mantle viscosity, and lower mantle viscosity to 70 km, 4×10^{20} Pa s, and 5×10^{22} Pa s. **(c)** RSL observations in the Bonaparte Gulf with GIA predictions using ANU model. A black dashed line corresponds to ice volume equivalent sea level**.** Gray lines are GIA predictions at elastic lithosphere, upper mantle viscosity, and lower mantle viscosity to 70 km, $(1, 4, 6, 9) \times 10^{20}$ Pa s, and $(0.5, 1, 5, 10) \times 10^{22}$ Pa s. A black line is GIA prediction at elastic lithosphere, upper mantle viscosity, and lower mantle viscosity to 70 km, 4×10^{20} Pa s, and 5×10^{22} Pa s.

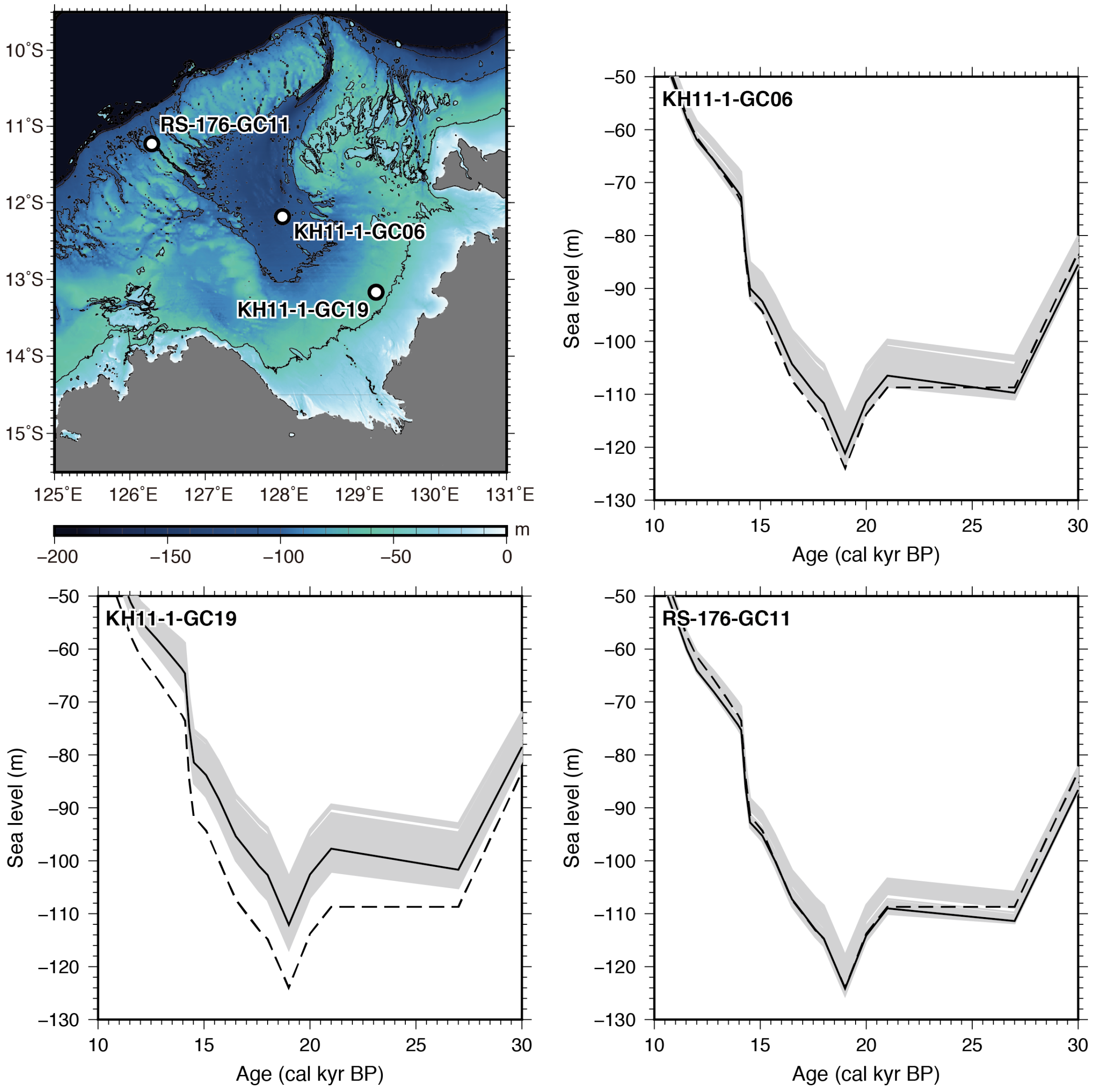

Figure S8 | GIA predictions of selected sits in the Bonaparte Gulf. A sea-level gradient due to local isostatic effect is occurred in the Bonaparte Gulf. Dashed lines correspond to ice volume equivalent sea level. Black lines are GIA predictions at elastic lithosphere of 70 km, upper mantle viscosity of 4×10^{20} Pa s, and lower mantle viscosity of 5×10^{22} Pa s. Gray lines are GIA predictions at elastic lithosphere of 70 km, upper mantle viscosity of $(1\text{–}9) \times 10^{20}$ Pa s, and lower mantle viscosity of $(0.5 - 0.9, 1.0\text{–}10) \times 10^{22}$ Pa s.

Table S1 | Locations, water depths and length of cores discussed in this study.

Core	Latitude S (deg. min.)	Longitude E (deg. min.)	Depth (m)	Length (cm)
GC03	11 55.628	128 09.269	111	336
GC06	12 11.038	128 01.513	120	583
GC07	12 06.190	128 06.088	115	504
GC08	12 05.004	128 10.181	114	500
GC09	11 59.431	128 12.269	112	268
GC10	12 05.342	128 16.849	106	460
GC11	12 15.490	128 24.607	108	380
GC13	12 40.039	128 47.017	96	311
GC14	12 45.495	128 52.326	88	67
GC19	13 10.197	129 16.034	67	146

Table S2 | Radiocarbon age results of KH11-1 cores using MatCal (Lougheed and Obrochta, 2016) for calibration analysis.

YAUT-: a laboratory number of the Single Stage Accelerator Mass Spectrometry at the University of Tokyo.

B274: a laboratory number of the Micro Analysis Laboratory Tandem Accelerator at the University of Tokyo.

N/A: the ages are excluded from age-depth model.

median mod: median calendar age obtained through age modeling

mod 2 sig lo: lower modeled calendar age range (2 sigma)

mod 2 sig hi: upper modeled calendar age range (2 sigma)

mod 1 sig lo: lower modeled calendar age range (1 sigma)

mod 1 sig hi: upper modeled calendar age range (1 sigma)

cal range lo 2: first calibrated calendar year 2 sigma lower bounds.

cal range hi 2: first calibrated calendar year 2 sigma upper bounds.

prob 2: probably of first calendar year range (limited to prob >=0.05)

Laboratory number	Core	Depth in core (cm)	14C age (yr BP)	median mod	mod 2 sig lo	mod 2 sig hi	mod 1 sig lo	mod 1 sig hi	cal range lo 2	cal range hi 2	prob 2	Material
YAUT-002708	GC03	37	15700±40	18570	18460	18680	18510	18640	18439	18705	0.95	unknown macrofossil
YAUT-002711	GC03	45	17060±50	20090	19940	20230	20000	20160	19903	20288	0.95	unknown macrofossil
YAUT-002709	GC03	60	17030±50	20150	20010	20240	20080	20210	19865	20248	0.95	*Spisula* sp.
YAUT-002710	GC03	74	17110±50	20230	20090	20320	20160	20280	19964	20352	0.95	*Spisula* sp.
YAUT-002707	GC03	88	17530±50	N/A	N/A	N/A	N/A	N/A	N/A	N/A	N/A	*Spisula* sp.
YAUT-025118	GC03	96	17090±50	20270	20150	20330	20210	20310	19940	20327	0.95	*Paphia undulata*

YAUT-002106	GC03	121	17170±120	20360	20220	20530	20290	20450	19924	20546	0.95	unknown macrofossil
YAUT-025101	GC03	215	17880±50	21090	20920	21290	20990	21200	20887	21324	0.95	Benthic foraminifera
YAUT-025104	GC03	260	17990±50	21250	21070	21360	21160	21330	21014	21481	0.95	Benthic foraminifera
YAUT-025105	GC03	296	17910±50	21300	21150	21360	21230	21350	20920	21365	0.96	Benthic foraminifera
YAUT-001729	GC06	70	15880±80	18740	18600	18870	18660	18810	18570	18898	0.95	*Spisula* sp.
YAUT-001730	GC06	74	16210±170	18950	18780	19040	18860	19010	18743	19518	0.95	*Spisula* sp.
YAUT-028832	GC06	86	16060±60	18990	18870	19040	18930	19030	18758	19043	0.96	Benthic foraminifera
YAUT-000828	GC06	132	16040±100	19040	18920	19130	18970	19100	18680	19135	0.95	*Spisula* sp.
YAUT-000818	GC06	169	16250±100	19160	19020	19390	19080	19280	18877	19415	0.95	*Spisula* sp.
YAUT-028833	GC06	200	16790±60	N/A	N/A	N/A	N/A	N/A	N/A	N/A	N/A	Benthic foraminifera
YAUT-001708	GC06	203	15840±140	N/A	N/A	N/A	N/A	N/A	N/A	N/A	N/A	*Spisula* sp.
YAUT-001725	GC06	215	16340±120	19340	19090	19520	19200	19450	18929	19538	0.95	*Turritella terebra*
YAUT-000823	GC06	229	16610±100	19570	19350	19820	19450	19720	19256	19871	0.95	*Paphia undulata*
YAUT-001721	GC06	270	16410±230	19700	19470	19890	19560	19840	18819	19900	0.95	*Anodontia Lucini*
YAUT-001719	GC06	280	16800±120	19850	19610	19960	19730	19920	19500	20094	0.95	*Spisula* sp.
YAUT-001710	GC06	290	16750±70	19900	19710	19970	19820	19950	19530	19967	0.95	*Spisula* sp.
YAUT-023933	GC06	299	17410±70	20480	20320	20590	20380	20550	20274	20725	0.95	Benthic foraminifera
YAUT-001706	GC06	320	17060±70	N/A	N/A	N/A	N/A	N/A	N/A	N/A	N/A	*Paphia undulata*
YAUT-000821	GC06	321	17410±100	20560	20390	20630	20500	20610	20208	20799	0.95	*Turritella terebra*
YAUT-001712	GC06	361	17340±70	20600	20450	20630	20540	20630	20184	20634	0.95	unknown macrofossil
YAUT-023934	GC06	400	18110±70	N/A	N/A	N/A	N/A	N/A	N/A	N/A	N/A	Benthic foraminifera

YAUT-003108	GC06	425	17690±80	20840	20640	21060	20730	20980	20599	21102	0.95	*Anadara* sp, *Paphia undulata*
YAUT-000811	GC06	463	18330±120	21720	21430	21990	21570	21850	21353	22058	0.95	*Anadara* sp.
YAUT-003111	GC06	465	19000±80	22470	22320	22640	22390	22550	22303	22661	0.95	*Abra* sp.
YAUT-000820	GC06	473	19800±120	23380	23060	23640	23170	23530	22995	23689	0.95	*Mimachlamys australia*
YAUT-000817	GC06	488	24510±170	28140	27840	28480	27960	28330	27792	28550	0.95	*Cucurbituls cymbium*
YAUT-003118	GC06	557	26460±140	30370	29940	30710	30150	30580	29837	30757	0.95	*Anadara* sp.
B274-C1110-20	GC06	559	26640±280	30670	30180	30950	30440	30830	29770	30991	0.95	unknown macrofossil
YAUT-023301	GC07	1	7530±40	N/A	N/A	N/A	N/A	N/A	N/A	N/A	N/A	Benthic foraminifera
B274-C111207-13	GC07	32	3260±70	3080	2910	3260	2990	3170	2883	3274	0.94	unknown coral
YAUT-023306	GC07	49	11870±40	N/A	N/A	N/A	N/A	N/A	N/A	N/A	N/A	Benthic foraminifera
YAUT-008618	GC07	90	3960±80	3960	3760	4150	3860	4060	3720	4187	0.95	unknown macrofossil
YAUT-023309	GC07	118	14830±60	N/A	N/A	N/A	N/A	N/A	N/A	N/A	N/A	unknown macrofossil
YAUT-023307	GC07	118	14290±40	16830	16630	17000	16710	16930	16592	17022	0.95	Benthic foraminifera
YAUT-008620	GC07	144	15410±90	18210	18000	18340	18100	18300	17972	18476	0.95	*Turritella terebra*
YAUT-008621	GC07	150	15300±90	18270	18090	18350	18190	18330	17884	18351	0.95	unknown macrofossil
YAUT-008623	GC07	155	14100±100	N/A	N/A	N/A	N/A	N/A	N/A	N/A	N/A	*Turritella terebra*
YAUT-008624	GC07	155	15820±120	18680	18460	18880	18560	18800	18417	18914	0.95	*Spisula* sp.
YAUT-021701	GC07	180	16030±50	18870	18770	18970	18820	18920	18750	18988	0.96	Benthic foraminifera
YAUT-021704	GC07	190	11890±40	N/A	N/A	N/A	N/A	N/A	N/A	N/A	N/A	unknown macrofossil
YAUT-021705	GC07	190	16440±50	19360	19200	19520	19270	19450	19179	19544	0.95	Benthic foraminifera
YAUT-021706	GC07	199	16680±50	19650	19510	19840	19570	19750	19484	19867	0.96	Benthic foraminifera

YAUT-016022	GC07	203	16750±80	19790	19610	19960	19690	19900	19519	19982	0.95	Benthic foraminifera
YAUT-023311	GC07	318	17550±60	20670	20510	20840	20590	20770	20490	20890	0.95	Benthic foraminifera
YAUT-021707	GC07	437	17520±60	20750	20590	20850	20660	20820	20457	20858	0.95	Benthic foraminifera
YAUT-010035	GC08	18	16870±60	N/A	N/A	N/A	N/A	N/A	N/A	N/A	N/A	*Spisula* sp.
YAUT-025119	GC08	58	16580±40	N/A	N/A	N/A	N/A	N/A	N/A	N/A	N/A	*Spisula* sp.
YAUT-003120	GC08	64	16990±110	N/A	N/A	N/A	N/A	N/A	N/A	N/A	N/A	*Turritella terebra*
YAUT-010037	GC08	64	16640±60	N/A	N/A	N/A	N/A	N/A	N/A	N/A	N/A	*Spisula* sp.
YAUT-025107	GC08	83	17150±50	N/A	N/A	N/A	N/A	N/A	N/A	N/A	N/A	Benthic foraminifera
YAUT-025121	GC08	83	16930±50	19940	19750	20100	19840	20030	19719	20126	0.95	*Spisula* sp.
YAUT-003121	GC08	94	16960±100	20060	19870	20230	19960	20150	19658	20251	0.95	*Paphia undulata*
YAUT-003122	GC08	104	17080±90	20190	20010	20370	20090	20290	19862	20401	0.95	*Spisula* sp.
YAUT-025109	GC08	119	17540±50	N/A	N/A	N/A	N/A	N/A	N/A	N/A	N/A	Benthic foraminifera
YAUT-003126	GC08	119	17100±80	N/A	N/A	N/A	N/A	N/A	N/A	N/A	N/A	unknown macrofossil
YAUT-025122	GC08	119	17260±50	20360	20180	20500	20270	20440	20124	20517	0.95	*Spisula* sp.
YAUT-025111	GC08	174	17850±50	21050	20880	21240	20950	21150	20853	21286	0.95	Benthic foraminifera
YAUT-010038	GC08	176	17800±70	21140	20940	21260	21040	21220	20748	21263	0.95	*Spisula* sp.
YAUT-003127	GC08	179	17820±130	21250	21020	21410	21150	21350	20655	21428	0.95	*Spisula* sp.
YAUT-025112	GC08	275	18050±50	21390	21210	21550	21300	21470	21079	21571	0.95	Benthic foraminifera
YAUT-025114	GC08	376	18090±50	21490	21330	21630	21400	21570	21151	21646	0.95	Benthic foraminifera
YAUT-004116	GC09	15	9600±40	N/A	N/A	N/A	N/A	N/A	N/A	N/A	N/A	unknown macrofossil
YAUT-004115	GC09	43	10130±50	N/A	N/A	N/A	N/A	N/A	N/A	N/A	N/A	unknown macrofossil

YAUT-004120	GC09	161	16600±50	19560	19400	19730	19470	19640	19361	19748	0.95	unknown macrofossil
YAUT-004121	GC09	166	16840±50	19830	19660	20000	19730	19940	19624	20028	0.95	unknown macrofossil
YAUT-004122	GC09	171	17180±110	20230	20000	20480	20090	20370	19956	20535	0.95	*Turritella terebra*
YAUT-028837	GC09	182	17320±60	20430	20230	20560	20340	20520	20179	20594	0.95	unknown macrofossil
YAUT-028834	GC09	182	17580±60	20670	20550	20820	20600	20750	20525	20925	0.95	Benthic foraminifera
YAUT-003108	GC09	185	17020±70	N/A	N/A	N/A	N/A	N/A	N/A	N/A	N/A	*Spisula* sp.
YAUT-004123	GC09	186	17490±100	20740	20600	20840	20660	20810	20322	20904	0.95	*Paphia undulata*
YAUT-004124	GC09	209	17760±110	20830	20700	20880	20770	20860	20626	21295	0.95	*Spisula* sp.
YAUT-003108	GC09	217	17320±70	N/A	N/A	N/A	N/A	N/A	N/A	N/A	N/A	*Spisula* sp.
YAUT-028829	GC09	218	17540±60	20850	20750	20880	20810	20880	20480	20880	0.95	Benthic foraminifera
B274-C111207-21	GC10	29	5470±80	5840	5670	5990	5750	5920	5640	6034	0.95	*Bufonaria rana*
YAUT-008619	GC10	82	18510±110	21950	21660	22240	21810	22120	21629	22282	0.95	*Turritella terebra*
YAUT-025124	GC10	174	19370±50	22840	22630	23010	22720	22930	22600	23042	0.95	unknown macrofossil
YAUT-010013	GC10	310	20010±80	23620	23390	23860	23500	23760	23352	23906	0.95	*Paphia undulata*
YAUT-025125	GC10	385	21170±60	25070	24760	25280	24940	25190	24698	25320	0.95	*Paphia undulata*
YAUT-010039	GC10	390	22010±90	25890	25740	26030	25810	25960	25711	26056	0.95	unknown macrofossil
YAUT-025127	GC11	57	14960±50	17740	17580	17900	17650	17830	17560	17924	0.95	unknown macrofossil
YAUT-023936	GC11	119	21810±80	25740	25570	25880	25650	25820	25552	25909	0.95	Benthic foraminifera
YAUT-023937	GC11	192	21890±80	25840	25700	25950	25770	25900	25623	25964	0.95	Benthic foraminifera
B274-C120806-6	GC13	10	3720±60	3650	3500	3800	3570	3740	3483	3816	0.95	Macrofossil
B274-C120806-5	GC13	17	13980±80	N/A	N/A	N/A	N/A	N/A	N/A	N/A	N/A	*Paphia undulata*

B274-C120806-4	GC13	75	14370±80	16940	16650	17180	16780	17070	16605	17227	0.95	*Semicassis bisulcata*
YAUT-001734	GC13	118	14060±130	N/A	N/A	N/A	N/A	N/A	N/A	N/A	N/A	unknown macrofossil
B274-C120806-3	GC13	164	14380±90	17070	16820	17260	16960	17190	16598	17284	0.95	unknown macrofossil
YAUT-002121	GC14	22	12490±120	13970	13720	14270	13840	14110	13645	14356	0.95	*Turritella terebra*
YAUT-002119	GC14	34	12930±150	14720	14220	15170	14410	15010	14146	15239	0.95	*Placamen gravescens*
YAUT-003732	GC19	13	610±30	N/A	N/A	N/A	N/A	N/A	N/A	N/A	N/A	unknown macrofossil
YAUT-003731	GC19	23	-900±20	N/A	N/A	N/A	N/A	N/A	N/A	N/A	N/A	unknown macrofossil
YAUT-003739	GC19	36	560±20	N/A	N/A	N/A	N/A	N/A	N/A	N/A	N/A	unknown macrofossil
YAUT-003905	GC19	84	11670±40	13160	13080	13260	13120	13220	13059	13289	0.95	unknown macrofossil
YAUT-004005	GC19	90	11320±40	13210	13130	13260	13170	13250	13082	13265	0.96	Peat
YAUT-004006	GC19	98	11570±40	13410	13310	13470	13350	13440	13302	13477	0.95	Peat

References

1. Lougheed, B. C. & Obrochta, S. P. MatCal: Open source Bayesian ^{14}C age calibration in MatLab. *Journal of Open Research Software* **4**, 1–4 (2016)
2. Whiteway, T. Australian Bathymetry and Topography Grid, June 2009. Scale 1:5000000. *Geoscience Australia Canberra*, (2009)
3. Yokoyama, Y., Lambeck, K., De Deckker, P., Johnston, P. & Fifield, L. K. Timing of the Last Glacial Maximum from observed sea-level minima. *Nature* **406**, 713–716 (2000)
4. Nicholas, W. A. et al. Pockmark development in the Petrel Sub-basin, Timor Sea, Northern Australia: Seabed habitat mapping in support of CO_2 storage assessments. *Continental Shelf Research* **83**, 129–142 (2014)
5. Lambeck, K., Rouby, H., Purcell, A., Sun, Y. & Sambridge, M. Sea level and global ice volumes from the Last Glacial Maximum to the Holocene. *Proceedings of the National Academy of Sciences* **111**, 15296–303 (2014)
6. Waelbroeck, C., Labeyrie, L., Michel, E., Duplessy, J.C., McManus, J.F., Lambeck, K., Balbn, E., Labracherie, M. Sea-level and deep water temperature changes derived from benthic foraminifera isotopic records. *Quaternary Science Reviews* **21**, 295–305 (2002)
